# Evaluation of prescribing practices and treatment failure for purulent skin and soft tissue infections in patients with obesity

**DOI:** 10.1017/ash.2024.441

**Published:** 2025-02-06

**Authors:** Mackenzie L. Miller, Destiny M. Hughson, Noah D. Blower, Andrew P. Jameson, Lisa E. Dumkow

**Affiliations:** 1 Department of Pharmacy, Trinity Health Grand Rapids, Grand Rapids, MI, USA; 2 Division of Infectious Diseases, Trinity Health Grand Rapids, Grand Rapids, MI, USA; 3 Department of Medicine, Michigan State College of Human Medicine, Grand Rapids, MI, USA

## Abstract

**Objective:**

Evaluate prescribing practices and risk factors for treatment failure in obese patients treated for purulent cellulitis with oral antibiotics in the outpatient setting.

**Design:**

Retrospective, multicenter, observational cohort.

**Setting:**

Emergency departments, primary care, and urgent care sites throughout Michigan.

**Patients:**

Adult patients with a body mass index of ≥ 30 kg/m^2^ who received ≥ 5 days of oral antibiotics for purulent cellulitis were included. Key exclusion criteria were chronic infections, antibiotic treatment within the past 30 days, and suspected polymicrobial infections.

**Methods:**

Obese patients receiving oral antibiotics for purulent cellulitis between February 1, 2020, and August 31, 2023, were assessed. The primary objective was to describe outpatient prescribing trends. Secondary objectives included comparing patient risk factors for treatment failure and safety outcomes between patients experiencing treatment success and those experiencing treatment failure.

**Results:**

Two hundred patients were included (Treatment success, n = 100; Treatment failure, n = 100). Patients received 11 antibiotic regimens with 26 dosing variations; 45.5% were inappropriately dosed. Sixty-seven percent of patients received MRSA-active therapy. Treatment failure was similar between those appropriately dosed (46.4%) versus under-dosed (54.4%) (*P* = 0.256), those receiving 5–7 days of therapy (47.1%) versus 10–14 days (54.4%) (*P* = 0.311), and those receiving MRSA-active therapy (52.2%) versus no MRSA therapy (45.5%) (*P* = 0.367). Patients treated with clindamycin were more likely to experience treatment failure (73.7% vs 47.5%, *P* = 0.030).

**Conclusions:**

Nearly half of antimicrobial regimens prescribed for outpatient treatment of cellulitis in patients with obesity were suboptimally prescribed. Opportunities exist to optimize agent selection, dosing, and duration of therapy in this population.

## Background

Skin and soft tissue infections (SSTIs), such as cellulitis, are among the most prevalent infections occurring in approximately 14 million individuals in the United States annually.^1^ Data suggests that obesity may increase a patient’s risk of contracting SSTIs and obese patients are at a higher risk of treatment failure.^2,3^

In the outpatient setting, suboptimal oral antimicrobial dosing and pharmacokinetic changes in patients with obesity may play a role in the poor outcomes observed in this population, as they may have altered volume of distribution, drug clearance, and half-life which can impact antibiotic exposure. Currently, there are a lack of robust pharmacokinetic and pharmacodynamic data available evaluating oral antimicrobial dose adjustments in obesity to account for these alterations.^4,5^ Treatment failure may lead to increased costs and hospitalizations for patients. Additionally, subtherapeutic levels of antibiotics could lead to the emergence of drug-resistant pathogens.^2^ This study aimed to assess antibiotic selection and treatment outcomes in patients with obesity with purulent cellulitis who received outpatient therapy to inform prescribing decisions and methods to minimize these risks.

## Methods

### Study design and setting

This was a retrospective, multicenter cohort study of obese patients who received oral antibiotic treatment for purulent cellulitis across Trinity Health Michigan outpatient locations between February 1, 2020, and August 31, 2023. This included emergency departments, primary care and urgent care sites. Patients aged 18 years or older who were prescribed five or more days of antibiotic therapy for the treatment of purulent cellulitis, with a creatinine clearance of at least 30 mL/minute, and a body mass index (BMI) of at least 30 kg/m^2^ were eligible for study inclusion. Patients were excluded if they were treated for SSTI within the past 30 days, had a chronic wound infection, suspected polymicrobial infection such as diabetic foot or post-operative infection, documented concern for osteomyelitis, bilateral cellulitis, were receiving chronic antibiotic prophylaxis, had incision and drainage (I&D) performed without surrounding cellulitis, or if they were pregnant. This study was reviewed by the Institutional Review Board and qualified for exemption of patient consent.

### Data collection and study endpoints

A report was generated from the electronic medical record (EMR) of patients diagnosed with purulent cellulitis according to the International Classification of Disease, Tenth Revision (ICD-10) diagnosis codes of L03.9 and L02 who were prescribed an oral antibiotic. This report was randomized using a random number generator (Microsoft Excel, Microsoft Corp., Redmond, WA) prior to screening patients for inclusion. Patients were then screened until the calculated sample size was met for both groups. Patient demographics were collected as well as treatment and infection characteristics. The primary objective of this analysis was to describe outpatient prescribing practices (drug(s), dose, and duration of therapy) in obese patients with purulent cellulitis treated with oral antibiotics. Secondary objectives included comparing prescribing trends in dosing and duration to currently available guidelines and assessing patient risk factors for treatment failure, and patient safety outcomes within 30 days of treatment between patients who had treatment success and those who had treatment failure. We defined appropriate antimicrobial dosing as the patient receiving at least the minimum recommended dose based on best-practice guidelines and standard dosing references (Table 1).^6^ Patients who received dual therapy regimens were considered to receive an appropriate dose if both agents were prescribed at least the minimum recommended dose. Appropriate treatment duration was defined as 5–7 days per the Infectious Diseases Society of America (IDSA) SSTI best practice guidelines.^7^ Treatment failure was defined as a recurrence of infection, change of therapy based on culture results, or need for re-treatment within 30 days. Safety outcomes compared between groups included outpatient re-visits, hospital admissions, *Clostridioides difficile* infections, and adverse drug reactions. Re-visit was defined as returning to the emergency department, a primary care office, or urgent care center with SSTI-related concerns.

**Table 1.** Minimum daily dose of antimicrobials^4,5^

Antibiotic	Minimum Appropriate Daily Dose
Amoxicillin/clavulanate	1500/375 mg
Cefuroxime	1000 mg
Cephalexin	2000 mg (every 6-hour dosing) 3000mg (every 8-hour dosing)
Clindamycin	1200 mg
Doxycycline	200 mg
Sulfamethoxazole/trimethoprim	3200/640 mg

### Statistical analysis

Statistical analysis was performed with SPSS version 22 software (IBM, Armonk, NY). A sample size of 200 patients was calculated to allow detection of a 10% difference between groups when considering patient risk factors for treatment failure, assuming an alpha of 0.05 and 80% power. Nominal variables were compared with the chi-square or Fisher’s exact test and interval data with student’s t-test or Mann-Whitney U test, as appropriate, based on the distribution of the data.

## Results

### Study population

**Figure 1.**
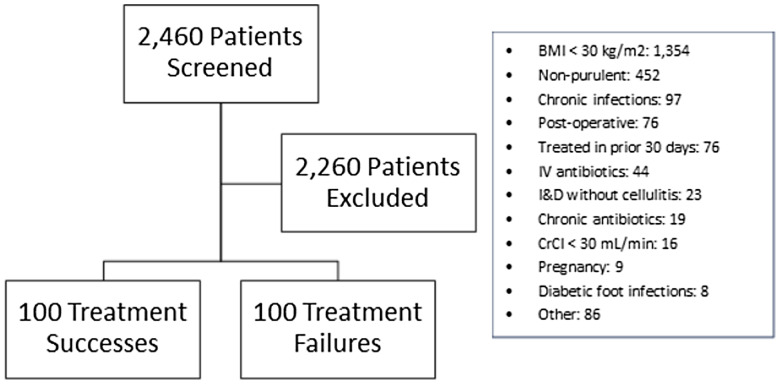
Patient population.

A total of 2,460 patients were screened for inclusion until 200 were found meeting eligibility criteria; 100 patients experiencing treatment success and 100 experiencing treatment failure were included (Figure 1). Patient characteristics are described in Table 2. Baseline characteristics were similar between groups except patients who experienced treatment failure were more likely to have a positive wound culture (21% vs 35%, *P* = 0.028). Of the 100 patients experiencing treatment failure, 70 required retreatment with oral antimicrobials, 18 required a change in prescribed oral antibiotic therapy to alternative therapy based on culture results, and 12 required hospital admission for IV antimicrobial therapy and/or surgical intervention. Of the 89 cultures obtained, *Staphylococcus aureus* was the most common pathogen isolated (MRSA and MSSA both 18%, respectively); 37% of cultures were negative. Primary care offices were the most common site of presentation (48.5%), followed by urgent care (35%), and emergency departments (16.5%). Treatment failure rates were similar among locations (55% vs 28% vs 17%, respectively; *P* = 0.102).

**Table 2.** Patient characteristics

	Treatment Success (n = 100)	Treatment Failure (n = 100)	*P* value
Average Age (years ± SD)	56 ± 14	56 ± 15	0.886
Female Sex, %	57	62	0.471
BMI Category, % • I (30 - < 35 kg/m^2^) • II (35 - < 40 kg/m^2^) • III (≥ 40 kg/m^2^)	45 25 30	36 27 37	0.405
Charlson Comorbidity Index, median [IQR]	2 [1 – 3]	2 [1 – 4]	0.473
Race, % • Caucasian • Hispanic • African American • American Indian	78 1 20 1	77 6 17 0	0.185
History of MRSA infection, %	11	6	0.205
I&D Performed, %	47	57	0.393
Wound Culture Collected, %	38	51	0.064
Wound Culture Positive, %	21	35	0.028

### Patient outcomes and risk factors for treatment failure

**Figure 2.**
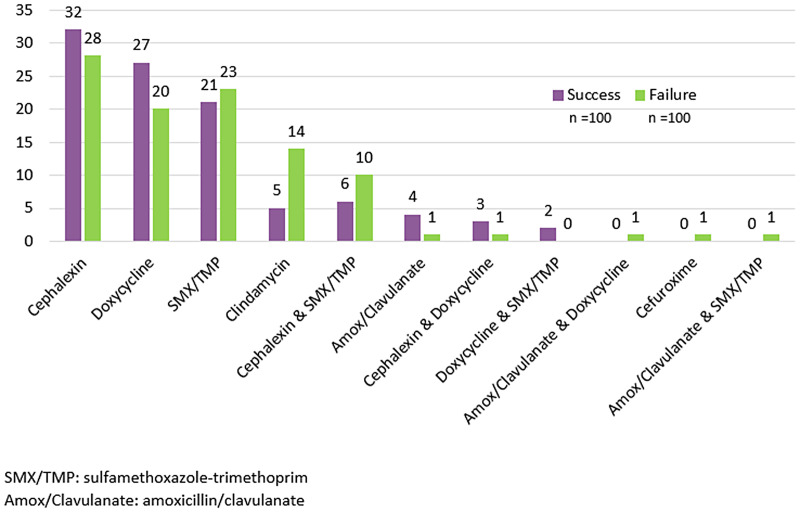
Antimicrobial agent(s) prescribed.

Eleven different antibiotic regimens were utilized for the treatment of purulent cellulitis (Figure 2) with 26 total dosing variations (Table 3). Cephalexin monotherapy was the most commonly prescribed regimen (30%), and 67% of patients received empiric MRSA-targeted therapy. Doxycycline was the most commonly prescribed MRSA-targeted therapy followed by sulfamethoxazole/trimethoprim (SMX/TMP) and clindamycin. Dual antimicrobial therapy was prescribed to 12% of patients. There were no instances of empiric linezolid prescribing. Ninety-five (47.5%) patients received an inappropriately dosed regimen, and 79 (39.5%) patients received a prolonged duration of therapy of 10–14 days.

**Table 3.** Antimicrobial regimens prescribed

Dosing Regimens Utilized	Success (n = 100)	Failure (n = 100)	Total (n = 200)	Appropriately dosed^*^
Monotherapy Regimens				
Amoxicillin-clavulanate 875 mg twice daily	4	1	5	X
Cefuroxime 500 mg three times daily	0	1	1	X
Cephalexin 500 mg twice daily	5	8	13	
Cephalexin 1000 mg twice daily	2	2	4	
Cephalexin 500 mg three times daily	7	6	13	
Cephalexin 1000 mg three times daily	0	3	3	X
Cephalexin 500 mg four times daily	18	9	27	X
Clindamycin 300 mg twice daily	0	2	2	
Clindamycin 150 mg three times daily	0	1	1	
Clindamycin 300 mg three times daily	1	4	5	
Clindamycin 300 mg four times daily	1	3	4	X
Clindamycin 450 mg three times daily	3	4	7	X
Doxycycline 100 mg twice daily	28	20	47	X
SMX/TMP 1 DS tablet twice daily	18	17	36	
SMX/TMP 2 DS tablets twice daily	3	5	8	X
Dual Therapy Regimens				
Amoxicillin-clavulanate 875 mg twice daily & doxycycline 100 mg twice daily	0	1	1	X
Amoxicillin-clavulanate 875 mg twice daily & SMX/TMP 1 DS tablet twice daily	0	1	1	
Cephalexin 500 mg twice daily & doxycycline 100 mg twice daily	0	1	1	
Cephalexin 500 mg four times daily & doxycycline 100 mg twice daily	2	0	2	X
Cephalexin 1000 mg two times daily & doxycycline 100 mg twice daily	0	1	1	
Cephalexin 500 mg twice daily & SMX/TMP 1 DS tablet twice daily	0	1	1	
Cephalexin 1000 mg twice daily & SMX/TMP 1 DS tablet twice daily	0	3	3	
Cephalexin 500 mg three times daily & SMX/TMP 1 DS tablet twice daily	1	1	2	
Cephalexin 500 mg four times daily & SMX/TMP 1 DS tablet twice daily	5	4	9	
Cephalexin 1000 mg four times daily & SMX/TMP 1 DS tablet twice daily	0	1	1	
Doxycycline 100 mg twice daily & SMX/TMP 1 DS tablet twice daily	2	0	2	

* Appropriately dosed defined as meeting minimum dosing recommendation in Table 1.

Treatment failure was similar between patients appropriately dosed (44.8%) versus under-dosed (55.7%) (*P* = 0.157), those who received 5–7 days of therapy (47.1%) versus 10–14 days (54.4%) (*P* = 0.311), those receiving I&D (47%) versus no abscess drainage (41%) (*P* = 0.367), and those who received MRSA-active therapy (52.2%) versus no MRSA therapy (45.5%) (*P* = 0.367). Patients empirically treated with clindamycin were more likely to experience treatment failure (73.7% vs 47.5%, *P* = 0.030) and had a higher rate of 30-day revisit (68.4% vs 47%, *P* = 0.075). There was no difference in reported adverse reactions between groups (2% in both groups, *P* = 1.0). No cases of *Clostridioides difficile* infection or nonadherence were reported in either group.

## Discussion

Across a Michigan health-system, various antimicrobial regimens and dosing strategies were utilized to treat patients with obesity who presented with purulent cellulitis in the outpatient setting. Our findings suggest significant variability in antibiotic prescribing for this patient population and lack of clear guidance recommending best agent(s) or dosing regimens. While obesity has been associated with altered pharmacokinetic and pharmacodynamic properties, the impact these changes may have on antimicrobial dosing is unclear. Drug volume of distribution is thought to be increased in obesity due to increased amounts of adipose and muscle tissue.^3^ An increased volume of distribution may impact antibiotic distribution in the body with lipophilic agents crossing into adipose tissue more easily than hydrophilic agents. Hydrophilic antibiotics, such as beta-lactams, may require increased doses to reach adequate concentrations.^3^ Additionally, renal clearance is thought to be increased in obesity due to an increase in renal mass, which may impact the rate at which antibiotics are cleared from the body.^3–5^ However, obesity is known to increase the risk of hypertension, diabetes, and other conditions that may conversely reduce renal function, making assessing the total impact on renal clearance complicated.^4^ Obesity has also been associated with slowed gastric emptying, which may hinder drug absorption, although data is not conclusive on this impact.^4^ Together, these characteristics suggest dose adjustments may be necessary in obesity to maintain sufficient antimicrobial concentrations to treat infection, although it remains unclear which agents may require increased doses and which may not need adjustment. For our more common agents, current literature recommends using higher doses of beta-lactams, SMX/TMP, and potentially clindamycin, with no dose adjustment for doxycycline.^3,4^ While we expected to observe most prescribers utilizing doses on the higher end of recommended ranges among this patient population due to potential kinetic changes, this was not commonly observed in our cohort. Antimicrobials in our study were largely underdosed and did not follow the best practice recommendations for dosing even in the non-obese population.

*Staphylococcus aureus* is the most common causative pathogen for purulent SSTI and consideration should be taken for local rates of MRSA when treating these infections.^8^ Rajendran et al. found in their analysis of uncomplicated abscesses that 70.4% of cultures grew *S. aureus* with 87.8% MRSA, similar to the findings of Moran et al. with 76% of abscess isolates with *S. aureus* with 59% MRSA.^9,10^ Our study similarly found that of 56 positive cultures, 57% were S. aureus, with 50% MRSA. Thus, an appropriate antimicrobial regimen for purulent cellulitis includes MRSA coverage, which is reflected in the IDSA best practice guideline.^7^ In our study, most patients did receive MRSA coverage, however, a significant proportion of patients did not. Despite the risk for MRSA, the most prescribed regimen was cephalexin monotherapy, which was followed by doxycycline and SMX/TMP monotherapies. We hypothesize that this may be due to provider comfort with prescribing cephalexin for common infections, its low cost, and overall favorable tolerability profile. Clindamycin was the fourth most commonly prescribed regimen; however, we observed a significantly higher rate of treatment failure with clindamycin compared to other regimens. This may be due to the various clindamycin dosing regimens utilized, with most patients prescribed doses lower than what is recommended for standard-weight patients.^11^ There have been no pharmacokinetic studies on clindamycin in obese patients to date and optimal dosing in this population is unknown.^4^ Additionally, national trends show an increase in *S. aureus* resistance to clindamycin, demonstrated by Alexander et al. with an average resistance rate of 27% from 2002 to 2008.^12^ Local resistance trends among our study sites’ isolates show a similarly concerning trend of resistance.

The pharmacokinetics of linezolid, an oral antibiotic with excellent MRSA activity, have been evaluated in obesity, with data suggesting that typical dosing led to no difference in exposure between obese and non-obese patients.^13^ This makes it a potentially attractive agent for treatment of purulent SSTI infections in obesity. While this pharmacokinetic data reflects intravenous linezolid, oral linezolid is highly bioavailable, allowing similar concentrations as the intravenous formulation.^14^ Despite this, linezolid was not utilized for any patient in this analysis. We suspect this may be due to several reasons including the historically higher cost, unfamiliarity of outpatient providers with the spectrum of activity and side effect profile, and the potential for drug interactions with commonly prescribed psychotropic medications. There likely exists significant opportunity to improve care of obese patients with purulent SSTI by using oral linezolid over less preferrable agents such as clindamycin in cases of allergy, intolerance, or history of treatment failure with other first-line therapies.

Finally, we observed significant variation in the duration of antimicrobial therapy prescribed. Patients were equally likely to receive prolonged (10–14 days) durations of therapy whether they experienced treatment failure or not. The IDSA recommends a 5-day course of therapy for patients with uncomplicated purulent cellulitis and extension if there is a lack of improvement by the end of the treatment course.^7^ Despite this recommendation, nearly 40% of patients in our study empirically received a treatment duration of 10–14 days. Obesity has been identified as a risk factor for recurrent lower extremity cellulitis and is associated with an increased risk of venous insufficiency, which may present similarly to a cellulitis with warmth and erythema.^15,16^ Thus, it may be more difficult to observe resolution of true cellulitis in obese patients presenting with chronic venous insufficiency and they may be at risk to receive repeated prolonged courses of antibiotics for suspected cellulitis.

This study did have limitations. The variability in prescribing observed was an unanticipated finding of the study. As such, with the numerous antibiotic regimens prescribed, a calculated sample size of 200 patients was likely insufficient to detect a difference in outcomes between the different agents and dosing regimens. Additionally, as a retrospective analysis this study was limited to what was documented in the EMR. Factors such as severity of infection and patient comorbidities may influence the prescribing of longer durations or dual antibiotic regimens, however, we were unable to perform matching or regression analyses due to the heterogeneity of prescribing. We saw a low incidence of documented adverse events and nonadherence, which may have been under reported or under documented. We additionally relied on appropriate provider diagnosis of SSTI, ICD-10 coding, culturing, and chart documentation to determine whether the SSTI was purulent. We were also limited by potential loss to follow up, as to meet our definition of treatment failure patients needed to have a re-visit for new antibiotics or hospital admission. Lastly, a large limitation of this study is the uncertainty of appropriate antimicrobial dosing in obesity. There are currently no best-practice guidelines for standard dosing in obesity, and while we used currently available guidelines and dosing references to assess appropriateness it is unclear what is truly most appropriate in this population. The lack of strong, evidence-based, guidance and significant prescribing variability observed in our cohort importantly show that more pharmacokinetic and outcomes studies are needed across antimicrobial classes to establish what dosing adjustments may be necessary to maximize treatment success while maintaining patient safety.

Antimicrobial prescribing for outpatient treatment of purulent cellulitis in patients with obesity was heterogenous across a Michigan health system. Nearly half of antimicrobial regimens were suboptimally prescribed. Opportunities exist to optimize agent selection, dosing, and duration of therapy in this population.

## Data Availability

The authors confirm that the data supporting the findings of this study are available within the article and/or its supplementary materials.

## References

[1] Fish DN. Skin and soft tissue infections. In: DiPiro JT , Yee GC , Posey L , Haines ST , Nolin TD , Ellingrod V , eds. Pharmacotherapy: A Pathophysiologic Approach. 12th ed. New York, NY: McGraw-Hill Education; 2022:chap 133.

[2] Charani E , Gharbi M , Frost G , Drumright L , Holmes A. Antimicrobial therapy in obesity: a multicentre cross-sectional study. J Antimicrob Chemother 2015;70:2906–2912. doi: 10.1093/jac/dkv189 26174720 PMC4566962

[3] Meng L , Mui E , Holubar MK , Deresinski SC. Comprehensive guidance for antibiotic dosing in obese adults: 2022 update. Pharmacotherapy 2023;43:226–246. doi: 10.1002/phar.2769 36703246

[4] Castro-Balado A , Varela-Rey I , Mejuto B , et al. Updated antimicrobial dosing recommendations for obese patients. Antimicrob Agents Chemother 2024;68:e0171923. doi: 10.1128/aac.01719-23 38526051 PMC11064535

[5] Polso AK , Lassiter JL , Nagel JL. Impact of hospital guideline for weight-based antimicrobial dosing in morbidly obese adults and comprehensive literature review. J Clin Pharm Ther 2014;39:584–608. doi: 10.1111/jcpt.12200 25203631

[6] Lexi-Drugs. UpToDate Lexidrug. UpToDate Inc. https://online.lexi.com. Accessed June 21, 2024.

[7] Stevens DL , Bisno AL , Chambers HF , et al. Practice guidelines for the diagnosis and management of skin and soft tissue infections: 2014 update by the Infectious Diseases Society of America. Clin Infect Dis 2014;59:147–159. doi: 10.1093/cid/ciu296 24947530

[8] Kaye KS , Petty LA , Shorr AF , Zilberberg MD. Current epidemiology, etiology, and burden of acute skin infections in the United States. Clin Infect Dis 2019;68:S193–S199. doi: 10.1093/cid/ciz002 30957165 PMC6452002

[9] Rajendran PM , Young D , Maurer T , et al. Randomized, double-blind, placebo-controlled trial of cephalexin for treatment of uncomplicated skin abscess in a population at risk for community-acquired methicillin-resistant *Staphylococcus aureus* infection. Antimicrob Agents Chemother 2007;51:4044–4048. doi: 10.1128/AAC.00377-07 17846141 PMC2151464

[10] Moran GJ , Krishnadasan A , Gorwitz RJ , et al. Methicillin-resistant *S. aureus* infections among patients in the emergency department. N Engl J Med 2006;355:666–74. doi: 10.1056/NEJMoa055356 16914702

[11] Clindamycin (Systemic). Lexi-Drugs. UpToDate Lexidrug. UpToDate Inc; 2024. https://online.lexi.com. Accessed June 21, 2024

[12] Alexander AJ , Richardson SE , Sharma A , Campisi P. The increasing prevalence of clindamycin resistance in *Staphylococcus aureus* isolates in children with head and neck abscesses. Can J Infect Dis Med Microbiol 2011;22:49–51. doi: 10.1155/2011/261519 22654925 PMC3142593

[13] Bhalodi AA , Papasavas PK , Tishler DS , Nicolau DP , Kuti JL. Pharmacokinetics of intravenous linezolid in moderately to morbidly obese adults. Antimicrob Agents Chemother 2013;57:1144–1149. doi: 10.1128/AAC.01453-12 23254421 PMC3591894

[14] Bouza E , Muñoz P. Linezolid: pharmacokinetic characteristics and clinical studies. Clin Microbiol Infect 2001:4:75–82. doi: 10.1046/j.1469-0691.2001.00061.x. 11688537

[15] Yosipovitch G , DeVore A , Dawn A. Obesity and the skin: skin physiology and skin manifestations of obesity. J Am Acad Dermatol 2007;56:901–16. doi: 10.1016/j.jaad.2006.12.004 17504714

[16] Karppelin M , Siljander T , Vuopio-Varkila J , et al. Factors predisposing to acute and recurrent bacterial non-necrotizing cellulitis in hospitalized patients: a prospective case-control study. Clin Microbiol Infect 2010;16:729–34. doi: 10.1111/8j.1469-0691.2009.02906.x. 19694769

